# Deforestation and predator species richness as potential environmental drivers for roadkill of wild water deer in South Korea

**DOI:** 10.3389/fvets.2025.1483563

**Published:** 2025-01-31

**Authors:** Obaidul Islam, Ryota Matsuyama, Kyung-Duk Min

**Affiliations:** ^1^Laboratory of Veterinary Epidemiology, College of Veterinary Medicine, Chungbuk National University, Cheongju, Republic of Korea; ^2^Veterinary Epidemiology Unit, Graduate School of Veterinary Medicine, Rakuno Gakuen University, Ebetsu, Hokkaido, Japan

**Keywords:** roadkill, predator, deforestation, highway, risk factors

## Abstract

**Introduction:**

The roadkill incidence of Korean water deer (*Hydropotes inermis argyropus*) has become a nationwide concern in recent years because of its contribution to biodiversity loss. Various environmental risk factors for the occurrence of roadkill events were found. However, there is a gap in observational studies focusing on the effects of deforestation and predator species richness on the roadkill, despite their plausible mechanisms. This study aimed to investigate the associations between water deer roadkill events and environmental risk factors in South Korea.

**Methods:**

We analyzed 1,986 roadkill events of water deer recorded on highway routes managed by the Korean National Transport Center from 2019 to 2021 as an outcome variable, and the values of environmental factors collated as explanatory variables. Multivariate logistic regression models were used to investigate these associations.

**Results:**

This study highlighted two main explanatory variables: predator species richness and deforestation, and the results indicate that higher deforestation level was associated with higher odds of the roadkill incidence, with an odds ratio of 1.15 [95% confidence interval (CI) = 1.07–1.25] from the ordinary model and 1.11 (95% credible interval = 1.03–1.21) from the spatial regression model. Conversely, predator species richness is negatively associated with the roadkill events, with an odds ratio of 0.75 (95% confidence interval = 0.69 to 0.80) from the ordinary regression model and 0.76 (95% credible interval = 0.66–0.86) from the spatial regression model.

**Conclusion:**

These findings suggest that conservational effort, such as preventing wildlife diversity and mitigating deforestation could reduce the incidence of water deer roadkill events.

## 1 Introduction

The Korean water deer (scientifically known as *Hydropotes inermis argyropus*) is an indigenous and native species in Korea that is categorized as vulnerable by the International Union for Conservation of Nature Red List (1) due to several factors, including poaching, restricted range, and relentless destruction of its natural habitat (2). In contrast, water deer are abundant in South Korea (3). Consequently, the increasing number of water deer killed by collisions with vehicles (hereafter roadkill) in South Korea has become a significant hazard to these animals. A previous study estimated that approximately 60,000 water deer die annually on Korean roads (4). Another study showed that the Gangwon province in South Korea had the highest number of roadkill accidents from 2004 to 2019, with water deer constituting the largest proportion (5). Roadkill is not only an environmental issue but also a public health issue. Data from various European countries, where such incidents are regularly documented, indicate that 2–5% of deer-related accidents typically result in human injury. Across continental Europe, it is estimated that approximately 300 people lose their lives and 30,000 sustain injuries annually due to collisions with hoofed wildlife (6). Furthermore, as wild animal carcasses decompose, they create ideal breeding grounds for tick-borne bacterial pathogens, attracting disease vectors that threaten nearby human populations (7). Additionally, there are significant financial costs associated with deer roadkill. In the United States, the National Highway Traffic Safety Administration reported that the damage from deer-vehicle crashes exceeded $52 million in the Ohio state in 1996 (8). More recent studies estimate this cost to reach as high as $1.1 billion annually (9), with average car repair expenses ranging from $648 in Michigan to $1,000–2,000 in Pennsylvania (10, 11).

Investigating risk factors is important for coping with roadkill, considering that they can inform government policy-making, serve as warnings for the public, and help identify risky areas or times for such accidents. Various environmental risk factors have been studied, most of which are related to animal movement. For instance, wildlife moves more frequently during the dry season when searching for food (12). Subsequently, greater movement would increase the contact probability between the car and the animal, which in turn would increase the risk of roadkill. In addition, climatic factors can influence the risk of roadkill. For instance, a previous study in South Korea reported a higher incidence of roadkill during the dry season (13). However, the impact of seasonality can vary by ecological context; in Brazil, roadkill tends to be more frequent during the rainy season. This is associated with increased food availability and breeding season, increased wildlife movement, and an increased probability of wildlife crossing highways and roads (14). Additionally, pasture and agricultural areas are positively correlated with higher roadkill rates (15), because many wild animals primarily obtain food from agricultural crops. Consequently, there has been an increase in the number of animals in agricultural areas (16), which has increased the frequency of roadkill incidents. The abundance of wildlife near roads is another risk factor for roadkill. Previous studies have found that a higher carnivore abundance results in higher carnivore roadkill values (17). In addition, non-environmental factors, such as traffic volume, are also risk factors for roadkill of wild animals. Research conducted in Taiwan identified high traffic volume, road conditions, and adjacent landscapes as the primary factors contributing to the occurrence of roadkill events (18).

On the other hand, the effects of deforestation and predator species richness on the roadkill have been under-studied, although there is an ecologically plausible mechanism. Deforestation could increase the risk of roadkill, because a previous study suggested that habitat disruption can lead to longer travel distances by increasing foraging efforts (19), which could subsequently increase roadkill event. Another study found that the lack of predatory species makes the prey more active (20), which could result in greater susceptibility to roadkill events. Despite the plausible nature of these associations, observational studies investigating the specific effects of these environmental factors on roadkill events are notably absent. We aim to address this gap by focusing on water deer roadkill events in South Korea as an example.

In this regard, there are two objectives in this study; (1) association between water deer roadkill and deforestation levels and (2) association between predator species richness and water deer roadkill in South Korea. By focusing on these objectives, we aimed to improve our knowledge of the ecological dynamics that lead to the roadkill of water deer and to develop more effective strategies for reducing the roadkill events in South Korea.

## 2 Materials and methods

### 2.1 Study design and unit of analysis

Associations of water deer roadkill events with deforestation and predator species richness were investigated using aggregate-level analysis. The locations of the national highways were acquired from the National Transport Center (21). Polygon–type spatial data were created from a 1 km buffer for the line–type spatial data of the highways. The polygon was divided into 1 km intervals, creating 3,391 highway segments. Each segment was used as a unit of analysis. Considering that the study period included 3 years (2019–2021), the number of study subjects was 10,173 (=3,391 × 3) highway segments. For each segment, the presence of roadkill events and values of environmental factors were allocated as the binary outcome (the distribution of frequency by highway segments were shown in Supplementary Table S1) and explanatory variables, respectively. Finally, the associations between the roadkills and the environmental factors were estimated using statistical models. In particular, we focused on two main explanatory variables: - richness of predator species and deforestation level, and considered the other variables as covariates.

### 2.2 Data acquisition and preprocessing

Geographical locations of wildlife roadkill events in highways were obtained from the National Institute of Ecology (NIE) (13). The point–type data included variables such as the time of occurrence (yearly) and, species of injured wildlife. Only roadkill events involving water deer were extracted, and the number of events for each highway segment was counted. Subsequently, the presence or absence of events in each segment were categorized as outcome variables.

**Figure 1:**
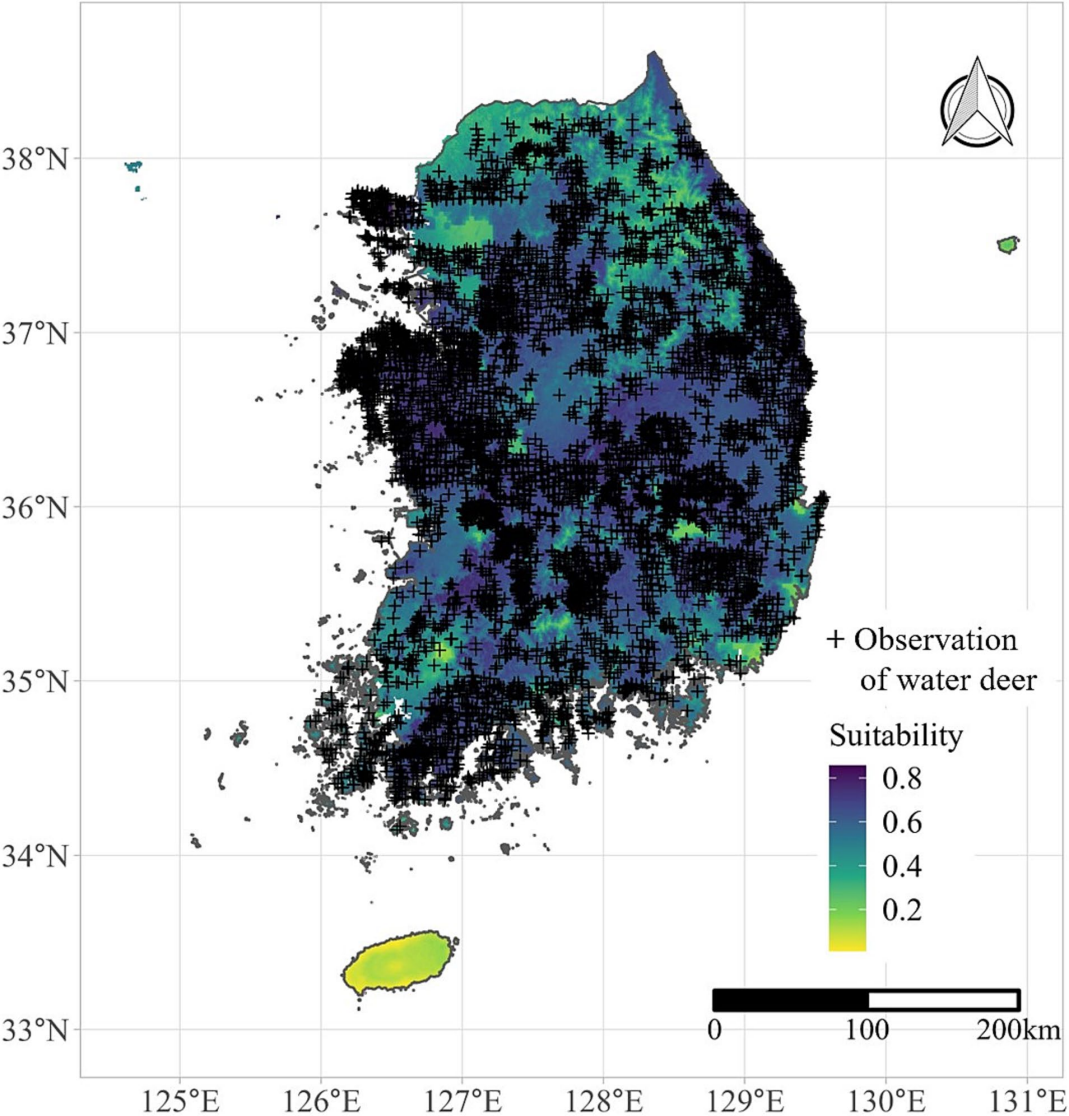
Estimated habitat suitability of water deer in South Korea. The estimated suitability of water deer indicates probability of water deer occurrence. It was derived using a species distribution modeling approach that incorporated observational data on water deer.

To create explanatory variables, locations of wildlife occurrence were obtained from the NIE, which conducts regular surveys of the natural environment. In the survey, investigators dispatched to local areas detected the existence of wildlife by identifying the geographical coordinates and species of the detected animals (22). The point data included all wildlife species, but we extracted only the data for water deer and its predator species. The locations of water deer occurrence were used to estimate the ecological suitability of each area for water deer using MaxEnt model, which incorporate climate factor, altitude and human population as explanatory variables. The estimated suitability was illustrated in Figure 1. The underlying principles and methodology for the estimation have been described previously (23). On the other hand, a variable of predator species richness was created by adding presence of target species which previous literature reported them as predator of deer species: *Haliaeetus pelagicus* (24), *Haliaeetus albicilla* (25), *Martes flavigula* (26), and *Bubo bubo* (27). Contrary to water deer, specific locations of the predator species were not accessible publicly because they are endangered species in South Korea. Considering only district-level presence can be acquired, we created the variable by each district. Subsequently, we designate the district-level variable to each highway segment (our study unit) by spatial join.

The other main explanatory variable was the deforestation level as obtained by Hansen et al. (28). The authors provided raster–type spatial data at the forestation level in 2000 and the presence of deforestation events from 2001 to 2021. The pixels in the forestation–level data had values from 0 to 100, indicating the probability of a forest. Using a threshold of 50, we categorized the pixels with and without a forest. Each study unit (highway segment) comprises multiple pixels. Accordingly, for each highway segment, we calculated the proportion of forest pixels and used it as an explanatory variable. Similarly, the proportion of deforestation was estimated. The pixels in the deforestation dataset had values ranging from 1 to 21, indicating deforestation events that occurred between 2001 and 2021. For each highway segment, we counted the number of deforestation events each year and divided this count by the total number of forested pixels. As a result, we obtained the proportion of pixels that experienced deforestation each year within each highway segment. The detailed methodology for creating the variables was described in a previous study (29).

Covariates, such as land use in urban areas and temperature, were also obtained. In terms of urban land use, satellite image data from NASA MODIS were used (30). As they provided raster–type spatial data with pixel values between 0 and 100, we employed a threshold of 50 to categorize the pixels with and without urban land use. Similar, to the forestation level, the proportion of land cover in each highway segment was calculated. The temperature data were obtained from the Automatic Synoptic Observation System operated by the Korea Meteorological Administration (31). As they provided the yearly mean temperature for each monitoring site, we spatially interpolated the variables to estimate temperature for each highway segment. Ordinary kriging was used in this study (32). There were other covariates in this study and the name of variables, their types of spatial data, and temporal resolutions are listed in Table 1.

**Table 1:** List of explanatory variables acquired and their spatial data types and temporal resolutions.

Variables	Type of spatial data	Temporal resolution
Occurrence locations of water deer	Point	NA*
Temperature	Points	Yearly
Predator species richness	Raster	NA*
Tree cover (%)	Raster	NA (in 2000)
Deforestation level (%)	Raster	Yearly
Urban area (%)	Raster	Yearly
Locations of highways	Lines	NA*
Roadkill events	Points	Yearly

*One value for the duration of the study.

### 2.3 Statistical analysis

A descriptive analysis was conducted to examine the differences in variables between highway segments with and without roadkill events. Student’s *t*-tests were used to examine the statistical significance of the differences because all explanatory variables (deforestation level, predator richness, suitability of water deer, temperature, year, treecover, and urban cover) were continuous. In addition, the geographical distribution of each variable was shown on maps.

Associations between outcomes and explanatory variables were investigated using a multivariable logistic regression model. Odds ratios (OR) and 95% confidence intervals (95% CI) were presented as model results. Additionally, a conditional autoregression model (CAR) was employed to incorporate spatial autocorrelation. Similarly, for the ordinary model, the OR and 95% credible interval (95% CrI) were shown as results. The multicollinearity of the explanatory variables was examined by examining the one-to-one association and suggesting a variance inflation factor (VIF).

Considering the objective of this study, which is investigating association of the roadkill events with deforestation and predator species richness, several covariates were also included in the models to adjust the association of main explanatory variables. The covariates included suitability of water deer, temperature, calendar year (2019, 2020, and 2021), urban land cover and forest land cover, which are either competing exposures or confounding factors (33). The higher suitability could indicate higher population of water deer, subsequently can act as competing factors (17). The land cover and climate factor can affect roadkill events by modulating wildlife behavior or human mobility (12–16). Temperature, for instance, can affect impact wildlife activity patterns, as most mammals showed the highest activity level around 15°C (34). The landcover and climate factor can also affect wildlife diversity, which indicate their role as confounding factor in an association between predator species richness and roadkill event. The calendar year was included as competing exposure, considering the possible effect of COVID-19 on reduced human mobility which can affect the roadkill event. The inclusion of calendar year can also adjust possible temporal autocorrelation. All explanatory variables and covariates were included in the models without any selection process.

### 2.4 Ethics statement

Ethical approval was not required for the study involving animals in accordance with local legislation and institutional requirements because only secondary data publicly available by Korean government were used.

## 3 Results

**Figure 2:**
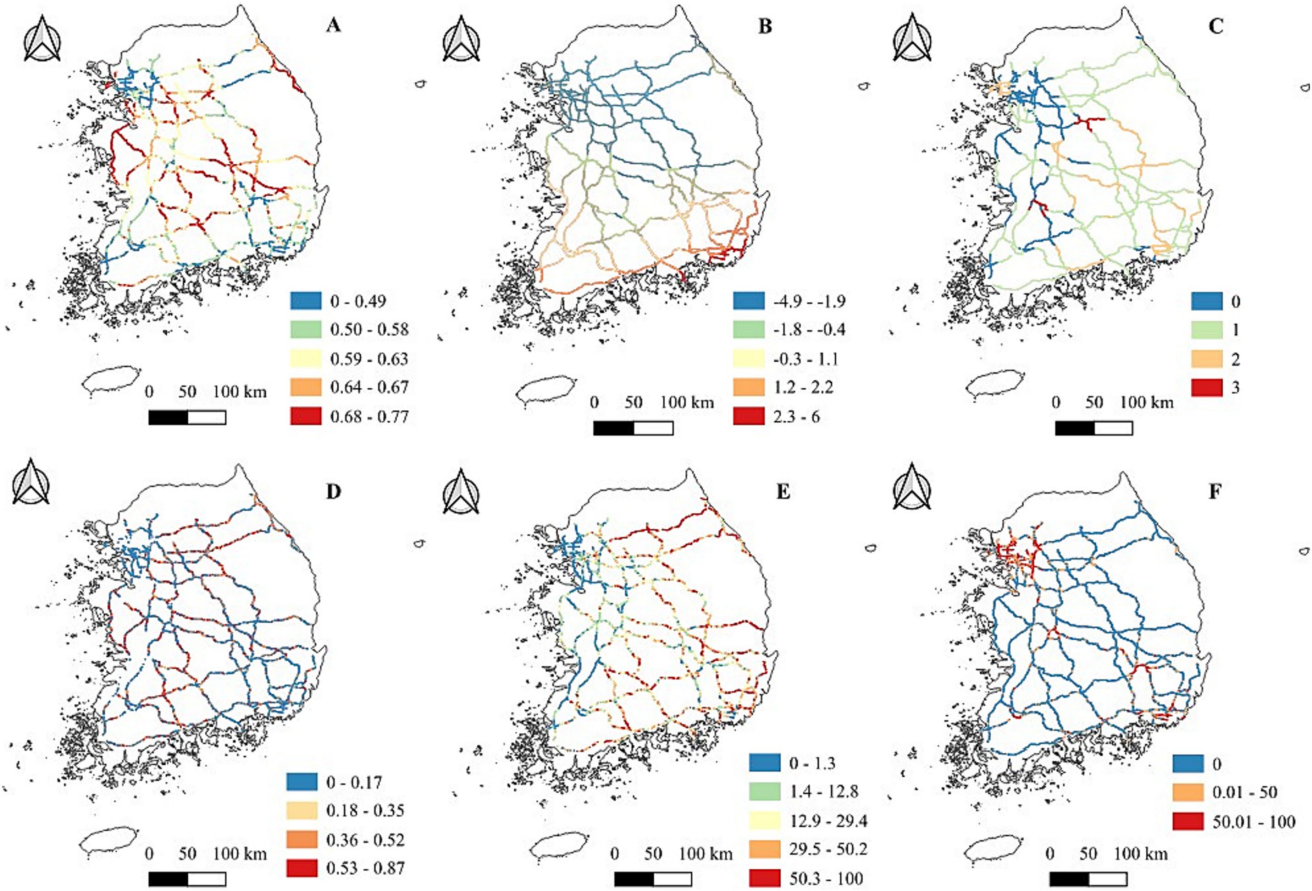
Spatial distribution of environmental variables in South Korean highways from 2019 to 2021. **(A)** Water deer habitat suitability, with values ranging from 0 to 1. Higher values indicate a greater probability of water deer presence. **(B)** Annual mean temperature. **(C)** Predator species richness, defined as the reported number of predator species. **(D)** Proportion of deforestation. **(E)** Proportion of tree land cover. **(F)** Proportion of urban land cover.

**Figure 3:**
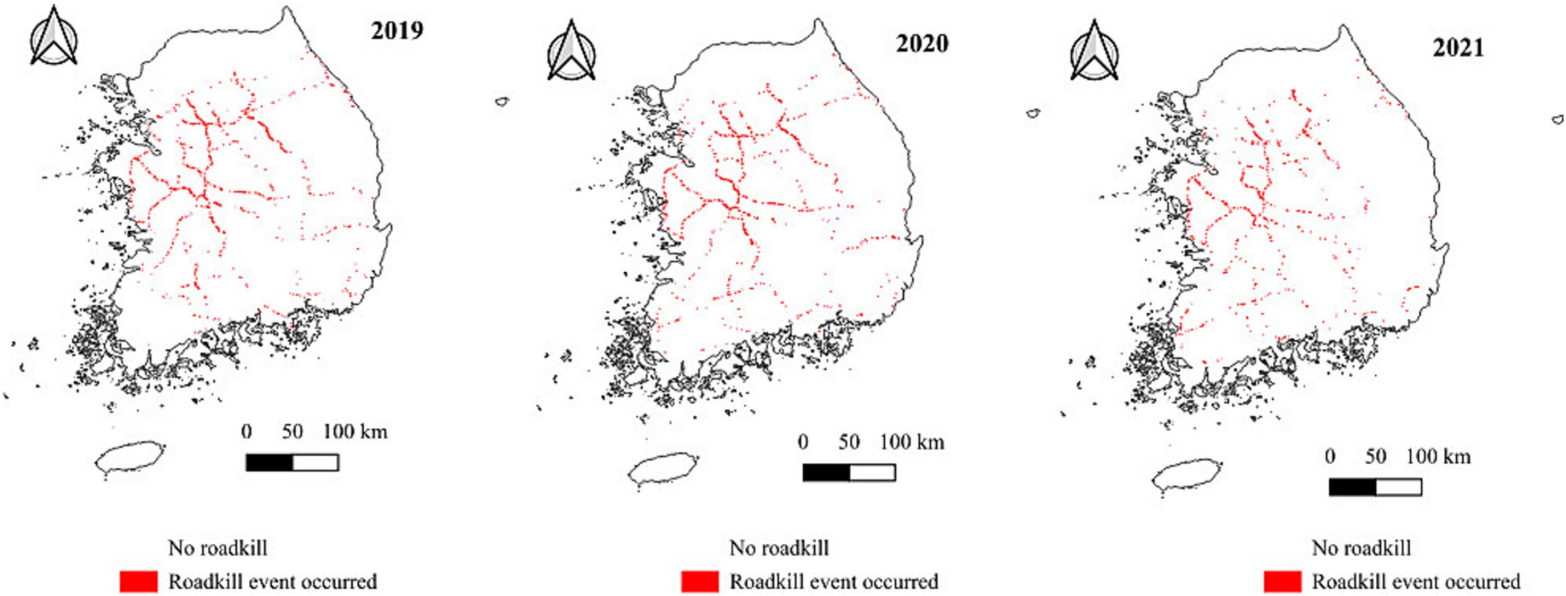
Location of water deer roadkill events in South Korean highways from 2019 to 2021.

Descriptive analysis revealed significant differences in most explanatory variables (Table 2) between segments with and without roadkill events. The suitability of water deer, deforestation level, and proportion of urban areas were significantly higher in highway segments with the roadkill events; however, temperature and tree cover were significantly higher in those without roadkill events. The distribution maps showed that the western and central regions of South Korean highways, which have higher levels of habitat suitability, more deforestation, and a higher proportion of urban areas, are where roadkill incidents of water deer are most common. In contrast, the southern highway region experiences fewer roadkill incidents, which have higher levels of environmental variables such as temperature, tree cover, and the richness of predator species (Figures 2, 3).

**Table 2:** Descriptive statistics of explanatory variables for highway segments with and without water deer roadkill events.

Variables	Mean ± sd		*p*-value (*t*-test)
	Highway segments with roadkill events (*N* = 1,986)	Highway segments without roadkill events (*N* = 8,088)	
Suitability of water deer	0.62 ± 0.09	0.58 ± 0.11	<0.001
Temperature	−0.15 ± 2.03	0.38 ± 2.28	<0.001
Predator species richness	0.80 ± 0.73	0.99 ± 0.73	<0.001
Tree cover (%)	0.21 ± 0.204	0.28 ± 0.26	0.010
Deforestation level (%)	0.16 ± 1.04	0.10 ± 0.57	<0.001
Urban area (%)	39.35 ± 36.42	28.98 ± 34.22	<0.001

**Figure 4:**
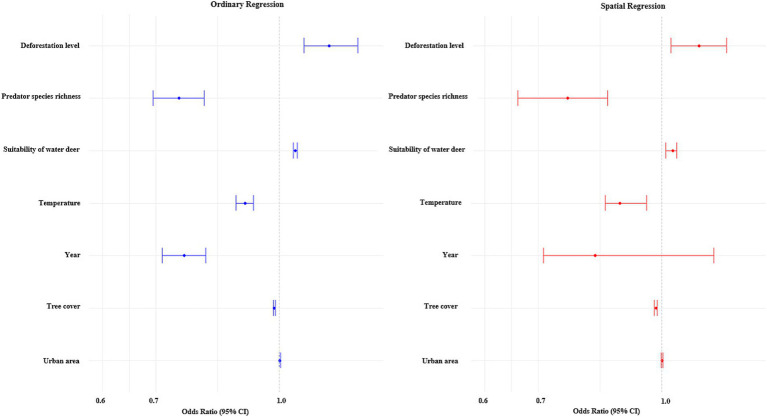
Estimated associations between water deer roadkill events and environmental variables. The left panel shows results from a multivariable ordinary logistic regression model, where outcome variable is whether there is at least one water deer roadkill event. The points and lines indicate point estimates and 95% confidence intervals for each explanatory variable. The right panel shows results from a multivariable spatial logistic regression model of which outcome variable is whether there is at least one water deer roadkill event. The points and lines indicate point estimates and 95% credible intervals of each explanatory variable.

The results of the ordinary and conditional autoregression models are presented in Figure 4. The direction and significance of the two models were identical. Deforestation tended to be higher in highway segments with roadkill events (OR [95% CI] from the ordinary model was 1.15 [1.07–1.25], and OR [95% CrI] from the CAR model was 1.11 [1.03–1.21]). In contrast, predator species richness showed significant negative associations (OR [95% CI] from the ordinary model was 0.75 [0.69–0.80] and OR [95% CrI] from the CAR model was 0.76 [0.66–0.86]). In terms of covariates, temperature, later years, and tree cover showed significant negative associations, although the effect size of tree cover was negligible. In contrast, the suitability for water deer and urban land cover showed significant positive associations. No multicollinearity was found because all VIF values were below 2.

## 4 Discussion

In the present study, we employed an ordinary (non-spatial) logistic regression model and CAR model that accounted for spatial autocorrelation, and the models included the calendar year, habitat suitability for water deer, and environmental factors surrounding the highways as covariates. The results showed significant positive and negative associations between deforestation and predator species richness, in both the non-spatial and CAR models, respectively.

This negative association between predator richness and water deer roadkill is a novel finding. As we hypothesized, the protective effect could be attribute to suppression of deer’s activity. A previous study suggested, predator species richness could affect foraging activity of prey species (35–37). A previous meta-analysis study (38) which suggested generalized effects of predator richness on suppressing prey species, also support our finding. However, it should be noticed that our hypothesized mechanism cannot be confirmed by current findings. Although the previous literature suggest that it is possible for the included predators hunting deer (24–27), it is still not confirmative that the predation effect is strong enough to representing the preventive effects on roadkill. Field experiment-based ecological studies are highly recommended to elaborate the underlying mechanism of current findings.

The positive association between deforestation levels and water deer roadkill incidents is another novel finding of this study. This association can be explained by the following two hypotheses. First, the cultivation of farmland or artificial structures leading to deforestation might increase traffic and human activity (i.e., movement by car) in the region, raising the likelihood of wildlife collisions (39). Additionally, deforestation can lead to forest fragmentation, which may increase the chances of collisions as animals move between forested areas. Water deer use forests and wetlands (40). If deforestation causes the frequent movement of water deer between forests and wetlands, the risk of collisions due to road crossings may increase. To test these hypotheses, it is crucial to elucidate the impact of deforestation on the ecology of water deer populations in affected areas.

We also examined the association between water deer roadkill and three other variables in the multivariate analysis: - habitat suitability, tree cover rate, and average temperature. It is quite natural that habitat suitability showed a strong positive association. The negative association of forest land cover can be explained by lower human activity or traffic volume. Considering that our multivariable models include water deer suitability as covariate, the higher forest land cover value in the models may solely indicate lower human mobility rather than water deer suitability. Because we did not include the traffic volume in the model due to lack of data accessibility, it can be proxy as the traffic volume. The negative association between the average annual temperature and water deer roadkill, could be caused by the relationship between vegetation density, temperature, and water deer roadkill. It has been reported that the temperature in areas with high vegetation density tends to be lower than that in areas with low vegetation (41, 42). Additionally, vegetation density as a food resource for water deer was positively correlated with water deer density. As a result, places with higher annual temperatures and lower vegetation had fewer water deer roadkills.

The effects of the coronavirus disease 2019 (COVID-19) pandemic since 2020 on wildlife-vehicle collisions have been controversial: - some studies have reported an increase, while others have not (43, 44). In this study, an inconclusive trend in roadkill events over time was found (Figure 4), although previous research by Park and Lee (45) demonstrated a reduction in wildlife-vehicle collisions in South Korea, primarily involving water deer, during the COVID-19 period. The reason for the difference between studies may be the timing of the observation. Park and Lee (45) focused only on the early phase of the COVID-19 pandemic (April–December 2020), whereas our study included roadkill data from 2021, which is the year when people became more accustomed to COVID-19. The discrepancy in results between the two studies implies that changes in traffic volume in response to COVID-19 may not have simple and consistent effects on the occurrence of roadkill events. Further studies are needed to elucidate the interaction between COVID-19 and water deer roadkill.

The present study has several limitations. First, we focused on the presence or absence of roadkill events rather than their number. Our model did not identify risk factors related to the frequency of roadkill events. Secondly, some variables evaluated in other studies were not included in the present study. For instance, the fine–scale data used by Kim and Hong (46) were not obtained because of the differences in the geographical scale of the present study. Additionally, this study focused only on highways, with fewer intersections than regular roads, and did not include variables such as road density used in Jang et al. (47). Third, individual host information was excluded. Many studies have suggested that certain periods are more prone to accidents based on sex and growth stage. For example, in deer, males during the breeding season and sub-adults during the dispersal period are more likely to be experience accidents (48). If there is a skewed sex ratio or a predominance of a particular growth stage in specific environments or years, a systematic error could be introduced into our study. Future research should establish a system for recording individual-level data, such as growth stage and sex, when wildlife roadkill occurs on highways.

In conclusion, this study revealed that the mechanisms underlying roadkill events involving water deer on highways in South Korea should consider the influence of two neglected environmental factors: predator richness and deforestation rate. Although the water deer population in South Korea is not considered vulnerable, it is an edge species with global conservation value. Furthermore, water deer can serve as an index species for understanding roadkill patterns, providing insights applicable to other species’ roadkill events. Additionally, from the perspective of wildlife management for mitigating traffic accidents, such as human-wildlife conflicts, it is crucial to establish an appropriate management plan for water deer roadkill. Continuous efforts to explore methods to reduce roadkill are necessary. Therefore, the effects of predator richness and deforestation, should be further elucidated.

## Data Availability

The data analyzed in this study is subject to the following licenses/restrictions: the datasets used and/or analyzed during the current study are available from the corresponding author on reasonable request. Requests to access these datasets should be directed to Kyung-Duk Min, kdmin@cbnu.ac.kr.
