# Supplementary material for: Deforestation and predator species richness as potential environmental drivers for roadkill of wild water deer in South Korea
Source: Front Vet Sci. 2025 Jan 31;12:1483563. doi: 10.3389/fvets.2025.1483563 (PMC11825762; doi:10.3389/fvets.2025.1483563)
Supplement: Supplementary file 1 [file Table_1.DOCX]

Table S1. Distribution of water deer roadkill frequency by highway segments

| Frequency of water deer roadkill events | Number of highway segments |
| --- | --- |
| 0 | 8,160 |
| 1 | 1,296 |
| 2 | 406 |
| 3 | 161 |
| 4 | 64 |
| 5 | 30 |
| 6 | 12 |
| 7 | 8 |
| 8 | 6 |
| 9 | 3 |
| 10 | 1 |
| 11 | 3 |
| 13 | 1 |
| 16 | 1 |
